# Traditional knowledge and cultural importance of *Gardenia erubescens* Stapf & Hutch. in Sudanian savanna of Burkina Faso

**DOI:** 10.1186/s13002-019-0305-4

**Published:** 2019-06-24

**Authors:** Korotimi Ouédraogo, Kangbéni Dimobe, Issouf Zerbo, Daniel Etongo, Alhassane Zare, Adjima Thiombiano

**Affiliations:** 1 Laboratory of Plant Biology and Ecology, University Ouaga I Pr Joseph Ki-Zerbo, UFR/SVT, 03 BP 7021, Ouagadougou 03, Burkina Faso; 2 Department of Environmental Science, University of Seychelles, Anse Royale, Seychelles

**Keywords:** Ethnobotany, Local knowledge, Conservation, Forest, Burkina Faso

## Abstract

**Background:**

Traditional knowledge (TK) on the different uses of under-valued fruit tree species including *Gardenia erubescens* Stapf & Hutch.—a plant species of least concern (LC) based on International Union for Conservation of Nature (IUCN) classification yet considered threatened due to overharvesting by a National Assessment in addition to 59 other species in Burkina Faso. This study aimed to collect and synthesize information on traditional knowledge and cultural importance of *G*. *erubescens*. This information will contribute to document traditional knowledge systems that are fast eroding due to the lack of transmission of the knowledge and will also highlight *G*. *erubescens* as a priority species for conservation given that this species is widely used among householders in rural areas in Burkina Faso.

**Methods:**

This study assesses TK on the uses and cultural importance (CI) of *G*. *erubescens*, among 514 randomly selected respondents across 15 villages bordering three community forest areas located in Eastern and Centre-Western Regions of Burkina Faso through face-to-face semi-structured interviews. Additionally, the uses and CI of *G*. *erubescens* were evaluated in relation to informant’s gender, ethnicity, generation, and location. Ethnobotanical indices (relative frequency of citation, relative use value, and CI) were computed using generalized linear models, Kruskal-Wallis, and Mann-Whitney tests.

**Results:**

Results indicated 30 specific uses of *G*. *erubescens* of which food values recorded the highest uses as reported by 58.97% respondents followed by medicine (17.22%) with a very low 0.23% for magical uses. Food and medicinal uses were the most important for women while men valued more the species for constructions, cultural, and magical uses. The fruit is the most preferred and marketable part of the plant while the leaves, fruits, roots, leafy twigs, and bark are mainly harvested for pharmacopeia and psycho-magical problems.

**Conclusions:**

Traditional knowledge on the uses of *G*. *erubescens* varied significantly in relation to gender, ethnicity, generations, and case study locations. Despite the importance of *G*. *erubescens* for food and other livelihood values, this specie is of LC to the IUCN; yet, a National Assessment considers it as threatened. The multiple uses of this specie based on TK systems for uses such as food, income, medicine, etc. is an indication that *G*. *erubescens* if sustainable managed could form an important safety net especially for rural households in Burkina Faso that are highly dependent on trees and forest resources.

## Background

Given the magnitude of the effects of climate variability and other global change polemics such as food insecurity and livelihood strategies, local knowledge on the uses of indigenous plants is invaluable to smallholder farmers, governments, non-governmental organizations (NGOs), policy makers, researchers, etc. in sub-Saharan Africa and the Sahel in particular [1, 2]. Indigenous plants provide fruits, seeds, tubers, and especially medicinal essences for the pharmacopeia, thus contributing to food, nutritional, and health benefits to local populations especially in rural areas [3]. In the Sahelian countries and Burkina Faso in particular, plant species are known for their multipurpose uses [2, 4, 5], providing products and services and therefore, these resources constitute an important component of local livelihoods, hence the need to evaluate the knowledge of the populations on plant species that have the potentials for food and health benefits [6].

This is the case of *Gardenia erubescens* Stapf & Hutch.—an indigenous species in Burkina Faso which is used locally for domestic energy supply, to generate cash income, for food, medicine, and crafts among other uses [7]. Its uses vary from one sociocultural group to another, generating a rich repertoire of traditional knowledge of this species. Although the overall IUCN threat category of *Gardenia erubescens* is least concern (LC), the species based on a National Assessment was identified alongside 59 other species as threatened as a result of overharvesting for multiple uses. Hence this species is considered threatened across its distribution range, especially in the North-Sudanian zone in Burkina Faso [8]. This species is considered extremely important for rural households given the significant income that can be generated from the sales of its fruits during seasons of food shortages or as a source of off-farm income. However, knowledge and exploitation of several products from *G*. *erubescens* remain unknown and confined to the hands of rural populations, hence the under-valuation of this species on all levels in our African countries where the species is present.

Patterns of local knowledge distribution can vary according to cultural [9] and socio-economic variables [10, 11]. For instance, men and women possess distinct ethnobotanical knowledge that is related to the different roles they play in local livelihoods [12]. To understand patterns of knowledge distribution within communities, quantitative methods have been used [10, 13, 14], including regression analysis [11, 15]. This is important, since the effects of one variable may depend on the levels of another one and carrying out analyses that do not allow for this could mask the “true” knowledge of local plant species thereby leading to conclusions that are misleading [14].

In Burkina Faso, previous studies have assessed the uses of some plant species, but they remain mostly global [5, 7, 16–18]. Other studies have addressed the ethnobotanical knowledge of local populations on the uses of *Vitellaria paradoxa* [19], *Bombax costatum* [18], *Lannea microcarpa* [20], and *Scleorocarya birrea* [20]. Belem et al. [21] studied the various uses of the trees out-of-forest and preferred by the Sanmatenga populations in Burkina Faso through ethnobotanical surveys. Ouédraogo et al.*,* [18] assessed the economic value of *Bombax costatum* in Burkina Faso. However, despite the number of studies on indigenous fruit tree species in Burkina Faso, there is no information regarding the specific ethnobotanical knowledge of several other underutilized species such as *G*. *erubescens*. Prized species for its fruits and its uses in the traditional pharmacopeia [22], *G*. *erubescens* contributes to the survival of rural populations during period of food shortages [7]. Empirical studies in Burkina Faso have documented changes in species richness that are directly linked to climate change, land use change, and over-exploitation. Within this context of continued forests and tree resource degradation and coupled with the adverse effects of climate change, data on economically valuable savanna tree species, including *G*. *erubescens*, are needed. This study aimed to collect and synthesize information on traditional knowledge and cultural importance of *G*. *erubescens*. This information will contribute to document traditional knowledge systems that are fast eroding due to change of generation and will also highlight *G*. *erubescens* as a priority species for conservation given that this species is among those that are considered threatened (locally) in Burkina Faso. More specifically, it is a question of determining the endogenous knowledge of uses of this species by the local populations of the classified forests of Boulon, Tapoa-Boopo, and Tiogo.

## Methods

### Study area

**Fig. 1:**
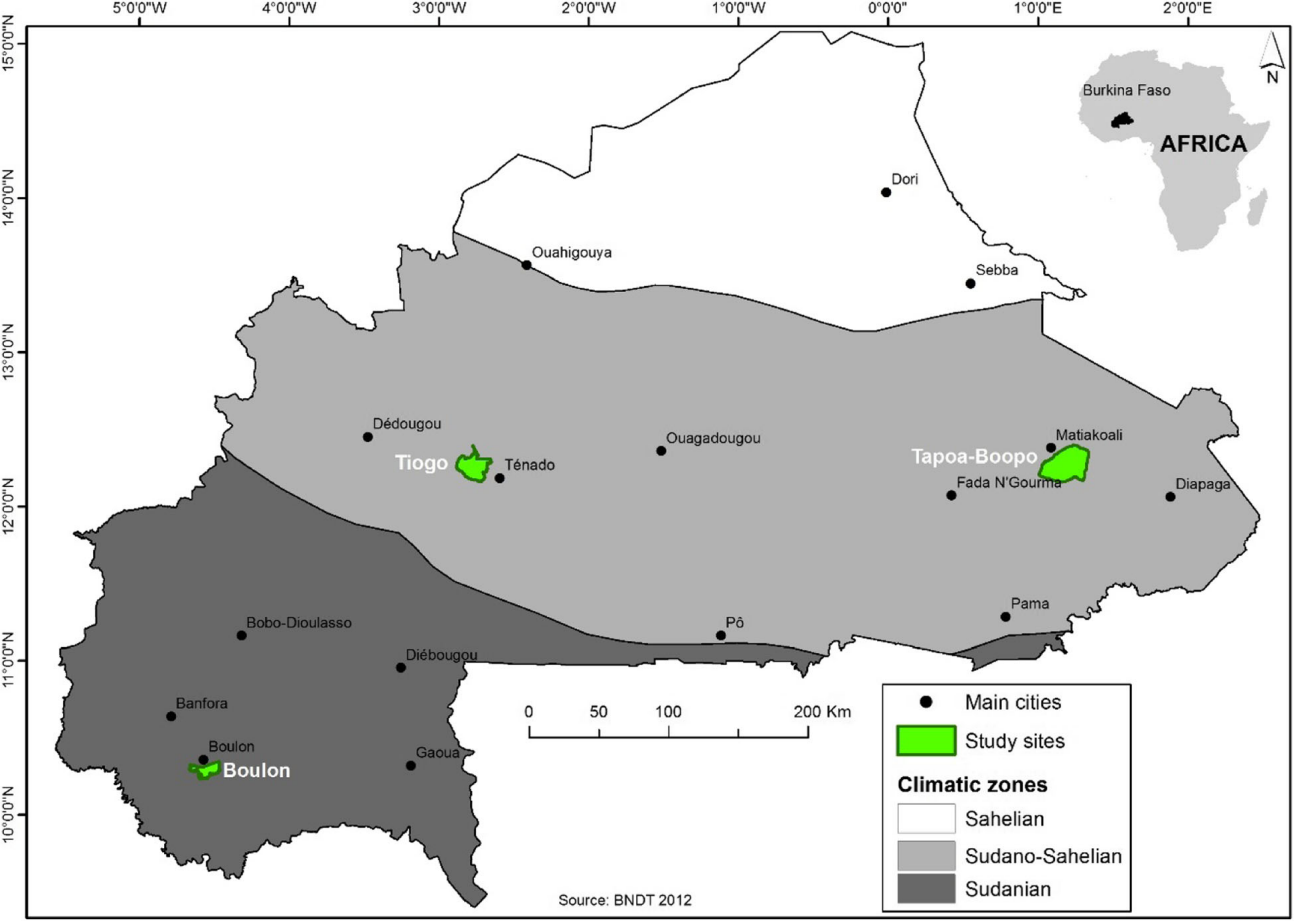
Location of the three-case study area in Burkina Faso all highlighted in green color

The study was conducted in 15 villages bordering three protected areas (PAs) located in the Cascades, Eastern, and West central Regions of Burkina Faso, namely Boulon, Tapoa-Boopo, and Tiogo (Fig. 1; Table 1). The description of the socio-political, governance, or biophysical contexts of the three forests areas are presented in Table 1. Boulon and Tiogo forests were classified by the colonial administration in 1955 and 1940, while that of Tapoa-Boopo was classified in the 80s [24]. In terms of climate, the forest of Boulon is located in the Sudanian zone while that of Tapoa-Boopo and Tiogo are located in the Sudano-Sahelian zone [23] as illustrated in Fig. 1. The subdivision of climatic zones in Burkina Faso is based on the annually rainfall amounts and the thermal regime. The Sahelian zone in the north of the country is characterized by an average annual rainfall lower than 600 mm, a high rate of evapotranspiration as well as high temperatures (> 30 °C), and a short rainy season (2 to 3 months) [8]. In the Sudano-Sahelian zone, the annual rainfall varies between 600 and 900 mm over 4- to 5-month period while the annual temperature ranges from 20 to 30 ° C. It comprises the most extensive climatic zone as it extends over all the central part of the country [8]. The Sudanian zone occupies the southern part of the country, where the rainy season lasts from 5 to 6 months with the level of rainfall attaining or even exceeding 1100 mm annually. This area is marked by low temperatures that range between 20 and 25 °C [8]. The current study is located in the last two climatic zones that experience a single rainy season that begins from the month of May to October with annual precipitation ranging between 600 and 1000 mm while the dry season occurs between the months of November and April. The mean annual temperature across the three sites is 35 °C; temperatures above 35 °C occur during peak period in the month of April [25]. The vegetation is a mosaic of shrubs and trees, woodland savannas, and grasslands. The main ethnic groups in the case study areas include Dogossin, Goin, Gourmantche, Gourounsi, Karaboro, Dioula, Mossi, and Fulani.

**Table 1:** Biophysical characteristics of Boulon, Tapoa-Boopo, and Tiogo forests [23]

Protected areas	Boulon	Tapoa-Boopo	Tiogo
Region	Cascades	Eastern	Central-West
Location	10° 15′–10° 22′ N; 4° 20′–4° 38′ W	12° 10′–12° 23′ N; 0° 58′–1° 13′ W	12° 10′–12° 25′ N; 2° 39′–2° 54′ W
Area (ha)	13,521.70	36,202.30	30,339
Climate	Sudanian	Sudano-Sahelian	Sudano-Sahelian
Annual rainfall (mm)	900–1100	600–900	600–900
Rainfall regime	Unimodal	Unimodal	Unimodal
Temperature range (°C)	20–25	20–30°	20–30°
Main vegetation	Dense dry forest, savannas	Tree and shrub savannas	Tree and shrub savannas

### Habitat and morphological descriptions of *Gardenia erubescens*

**Fig. 2:**
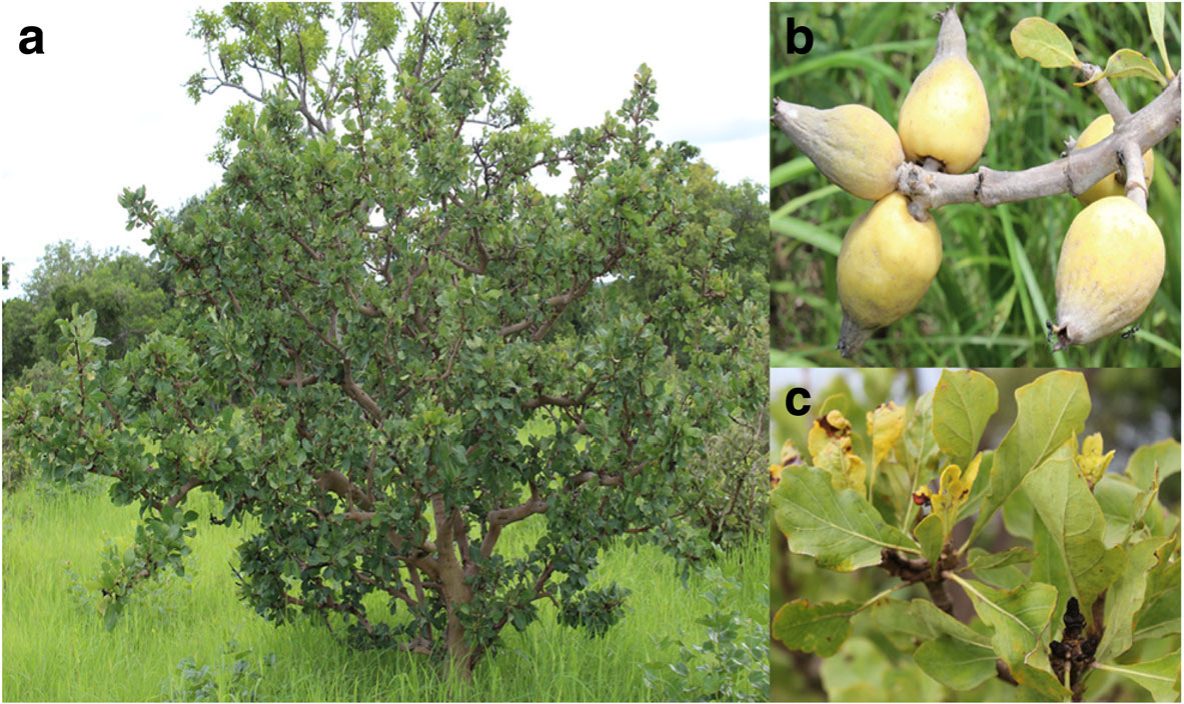
Tree specimen (**a**), fruits (**b**), and leaves (**c**) of *G*. *erubescens*

*Gardenia erubescens* Stapf & Hutch is a shrub species with a maximum height of 3 m above the ground (Fig. 2). It belongs to the genus Gardenia of flowering plants in the coffee family called Rubiaceae. This species is widely spread in tropical Africa, from Senegal to Sudan and Uganda. In Burkina Faso, the species is dominantly found in Sudanian zone [8]. The wood of the species is yellow, hard, and compact. The plant is sometimes gathered from the wild for local use as food, medicine, and as construction materials. The fruits are yellow and are used in sauces and soups (Fig. 2). The pale yellow, ellipsoid fruit can be 3–5-cm long. The leaves are opposite or in whorls of three or four, 5–50-cm long and 3–25-cm broad. They are dark green and glossy with a leathery texture. The flowers are solitary or in small clusters, either white, or pale yellow, with a tubular-based corolla with 5–12 lobes (petals) from 5 to 12 cm diameter. In Burkina Faso, *G*. *erubescens* is found in all three climatic zones but with great differences regarding its distribution and abundance [8].

### Data collection

The village authorities and their representatives were contacted, and the aim of the research was explained to get their consent. A consent form was part of the questionnaire for informants to understand the aim of the research and give their consent before engaging in the interview process. A pilot study (pre-test) was conducted in June 2016 to test the clarity of the questionnaire, determine suitability of the participants, and to determine the time required to complete a survey. The pre-test was also used to determine the number of respondents and to achieve this objective, a random sample of 40 people were selected for the pre-test. In order for the sample size to be representative, the number of respondents was calculated using the following formula [26]:

$$ N=\frac{U_{1-\alpha /2}^2\ast p\left(1-p\right)}{d^2} $$

Where *N* is the total number of people surveyed; *U* is the value of the random normal variable for a probability value of *α*; *U* = 1.96 if *α* = 0.05; *p* is the proportion of respondents who used or knew the species; and *d* is the margin error.

The pre-test revealed that 72, 80, and 78% of the respondents in Boulon, Tapoa-Boopo, and Tiogo, respectively, know and use *G*. *erubescens* (Fig. 2). A sample size of 159 (Boulon), 173 (Tapoa-Boopo), and 182 (Tiogo) people was determined for a 6% margin of error (*d*) which is acceptable in statistics.

Respondents were classified as young people (below 40 years) and old people (above 40 years) as described by Goudégnon et al. [9]. Both groups were considered to represent two generations and finally, the aspect of gender (men and women) and location was also considered. Ethnobotanical surveys were conducted in 15 villages across the three study sites in the PAs of Boulon, Tapoa-Boopo, and Tiogo using face-to-face individual interviews to assess the traditional knowledge and uses of *G*. *erubescens* based on 514 randomly selected informants. Direct field observation was used to complement information gathered from the informants (see Table 2). The surveys were conducted between the months of July to September 2016. Data collection included demographic information (age, gender, and ethnic groups), ethnobotanical knowledge of *G*. *erubescens* (vernacular name, specific use per plant part, categories of use), and the real use of the species. The real use of the species was determined by asking the informant to score the use-categories based on the importance of their actual uses. The score varied from 3 (high use) to 0 (not used) with score 2 for “moderate use” and score 1 for “low use” [2].

**Table 2:** Socio-demographic characteristics (ethnic group, age category, and gender) of informants and local names of *G*. *erubescens*

Ethnic group	Gender	Young	Old	Proportion of total respondents (%)	Local name
Dioula	F	4	4	3	Bure musso, Blé, Glé
	M	1	5		
Dogossin	F	3	5	4	Lalakô
	M	7	8		
Karaboro	F	6	4	6	Inchran chio
	M	8	15		
Goin	F	9	12	9	Blahou
	M	1	22		
Fulani	F	18	9	11	Dihanli, soubahé
	M	12	17		
Mossi	F	10	18	13	Subduga
	M	10	29		
Gourmantché	F	14	40	24	Bounasobou
	M	15	52		
Gourounsi	F	32	44	30	Kanton
	M	31	47		
Proportion of total respondents (%)		35	65	100	

*F* female, *M* male

**Fig. 3:**
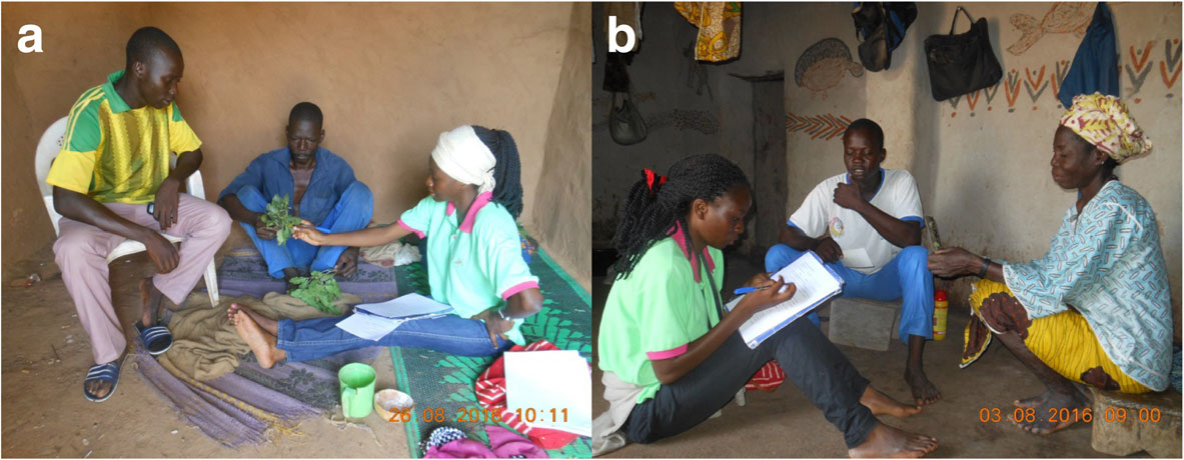
Example of an interview session with plant leaves as specimen for identification with a male informant (**a**); an interview session with a female informant (**b**). The third person in each case is a translator

Specimens of *G*. *erubescens*, in our case the leaves and fruits of the plant, were presented to interviewees (Fig. 3) to ensure the presence of the species in that village. Interviews were conducted in four local languages (Dioula, Mooré, Gourounsi, and Goulmancéma) that are commonly spoken in the study areas. A research assistant who understood these languages acted as a translator and each interview session lasted between 1 and 2 h.

### Data analysis

#### Knowledge on the uses of *G*. *erubescens*

To check if the information gathered was both relevant and scientifically valid, a number of quantitative ethnobotanical methods was applied to the collected data.

The relative frequency of citation (number of times a use was reported) for each use-category of *G*. *erubescens* was first computed using the following formula based on the fidelity level [27]:

$$ \mathrm{FL}\left(\%\right)=100\ast n/N $$

where *n* is the number of informants who mentioned a specific use and *N* is the total number of informants.

To assess the knowledge on uses of *G*. *erubescens*, the relative use value (UV) was used [2]. UV was calculated using the following formula:

$$ \mathrm{UV}=\sum {U}_i/N $$

where *Ui* is the number of uses mentioned by informant *i* and *N* is the total number of informants interviewed.

UV stands as a mean of the number of uses reported by informant *i* and calculated by use-category [9]. Thus, the number of uses was calculated by use-category and aggregated to obtain the total number of uses reported per respondent.

Seven categories of uses were identified: medicine, food, firewood, construction, culture, fodder, and magic. Because the number of uses reported by informant *i* for a given use-category is a count data, a generalized linear model (GLM) with Poisson error distribution [28] was applied to assess variation of UV with respect to sites, ethnic group, generation, and gender. The full model (main effect with interaction) was constructed first, then the effect of each factor (sites, generation, ethnic group, gender, and their interaction) was assessed using the chi-square test with the *anova* function in the R statistical software.

#### Cultural importance of *G*. *erubescens*

The cultural importance of use-category of *G*. *erubescens* was assessed using the importance index (IP) adapted from Hoeuhanou et al. [29]:

$$ \mathrm{IP}=\sum \limits_{uc=1}^{n_{uc}}{IP}_{uc}=\sum \limits_{uc=1}^{n_{uc}}\sum \limits_{i=1}^n{S}_{i, uc}/N $$

*Si,uc* is the score of importance attributed by informant *i* for the use-category *uc* and *nuc* is the number of use-category. *IP* is the overall importance value of the species and *IPuc* is the importance value of the use-category *uc* of the species.

Kruskal-Wallis and Mann-Whitney tests were performed on the IP to test for significant differences in the CI of the use categories and the plant parts between study sites, ethnic groups, generations, and gender.

All the statistical analyzes were performed in the R-3.3.2 [30] and the threshold of significance of the tests was fixed at 5%. GLMs were adjusted with the *MASS* package. The Kruskal-Wallis tests were carried out with the *agricolae* package [31].

## Results

### Demographic information and traditional knowledge on the uses of *G*. *erubescens*

A total of 514 people belonging to eight ethnic groups were interviewed to evaluate their TK and uses of *G*. *erubescens.* This tree species was identified by nine different local names among the eight ethnic groups present in the three case study areas (Table 2). Despite differences in local names of *G*. *erubescens* from one ethnic group to another, all of these names referred to the same species. In terms of its uses, a total of seven use categories were identified by the informants of which the two most reported uses were for food and medicine corresponding to 58.97% and 17.22% respectively and a minuscule percentage (0.23%) for magical uses. Six plant parts of the species were used locally, and many recipes were recorded for 30 different uses in addition to the treatment of several diseases (Appendix). The roots and leaves are the plant parts that are mostly utilized for traditional medicine to treat different types of diseases and as such, the leaves and roots are commonly harvested in addition to debarking for decoration purposes.

Furthermore, the number of uses reported per plant part were as follows: leaves (ten uses), roots (nine uses), young twigs (seven uses), and fruits (seven uses). The bark of the plant was involved in four uses locally, whereas three uses were reported for the wood from the plant (Appendix). Some of the uses of the different parts were reported by more than 80% of the respondents (Appendix).

The overall use value (UV) of *G. erubescens* varied significantly only between the eight ethnic groups given that *p* < 0.05 (see Table 3). Considering the analysis at the level of use-category, the results of the GLM indicated that the UV of *G*. *erubescens* for food, fodder, and magical uses was similar among the study sites, ethnic groups, and generation (*p* > 0.05) while that for medicinal use varied greatly between the study sites, ethnic groups, and generation because *p* < 0.05 (Table 3). The UV for cultural purposes differed significantly only between ethnic groups and generation (*p* < 0.05), whereas there was a significant relationship between energy use of *G*. *erubescens* and study sites, ethnic groups, and gender given that *p* < 0.05 (Table 3). Construction use varied significantly only among the study sites. However, there was no significant interaction between these factors.

**Table 3:** Influence of socio-demographic factors on use value

Use-category	Site (S)	Ethnic (E)	Generation (G)	Gender (A)	S x E	E x G	G x S	A x G
Food	0.874 ^ns^	0.9991 ^ns^	0.908 ^ns^	0.850 ^ns^	0.999 ^ns^	0.999 ^ns^	0.958 ^ns^	0.946 ^ns^
Medicine	< 0.001**	< 0.001**	< 0.001**	0.179 ^ns^	0.675 ^ns^	0.793 ^ns^	0.721 ^ns^	0.583 ^ns^
Construction	0.0169 *	0.3299 ^ns^	0.3964 ^ns^	0.343 ^ns^	0.638 ^ns^	0.895 ^ns^	0.247 ^ns^	0.386 ^ns^
Fodder	0.1128 ^ns^	0.8032 ^ns^	0.671 ^ns^	0.905 ^ns^	1.000 ^ns^	0.989 ^ns^	1.000 ^ns^	0.0849 ^ns^
Energy	< 0.0001***	< 0.0001***	0.323 ^ns^	0.045*	0.596 ^ns^	0.688 ^ns^	0.165 ^ns^	0.707 ^ns^
Cultural	< 0.0001***	< 0.0001***	0.323 ^ns^	0.165 ^ns^	0.989 ^ns^	0.227 ^ns^	0.100 ^ns^	0.190 ^ns^
Magic	0.112 ^ns^	0.6703 ^ns^	0.186 ^ns^	0.117 ^ns^	1.000 ^ns^	1.000 ^ns^	1.000 ^ns^	0.999 ^ns^
Overall use	0.055 ^ns^	0.020 *	0.574 ^ns^	0.879 ^ns^	0.452 ^ns^	0.767 ^ns^	0.377 ^ns^	0.403 ^ns^

*** = *p* < 0.0001; ** = *p* < 0.001; * = *p* < 0.01; ns = *p* > 0.05

**Fig. 4:**
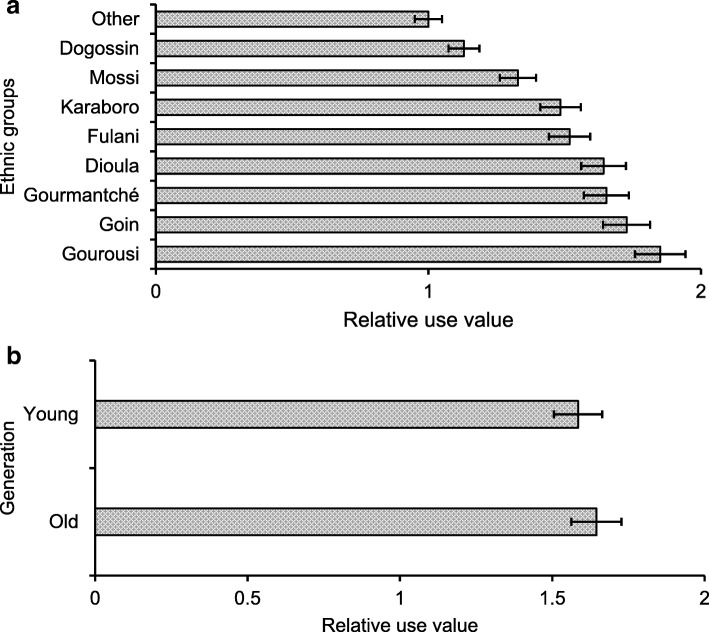
Total use value of *G*. *erubescens* by ethnic group (**a**) and generation (**b**)

Among the ethnic groups considered in this study, the Gourounsi, Goin, Gourmantché, and Dioula represent the ethnic groups that use *G*. *erubescens* the most in the study area (Fig. 4a). As for the generations, the old persons (age ≥ 40 years) reported more uses than the young (Fig. 4b).

**Fig. 5:**
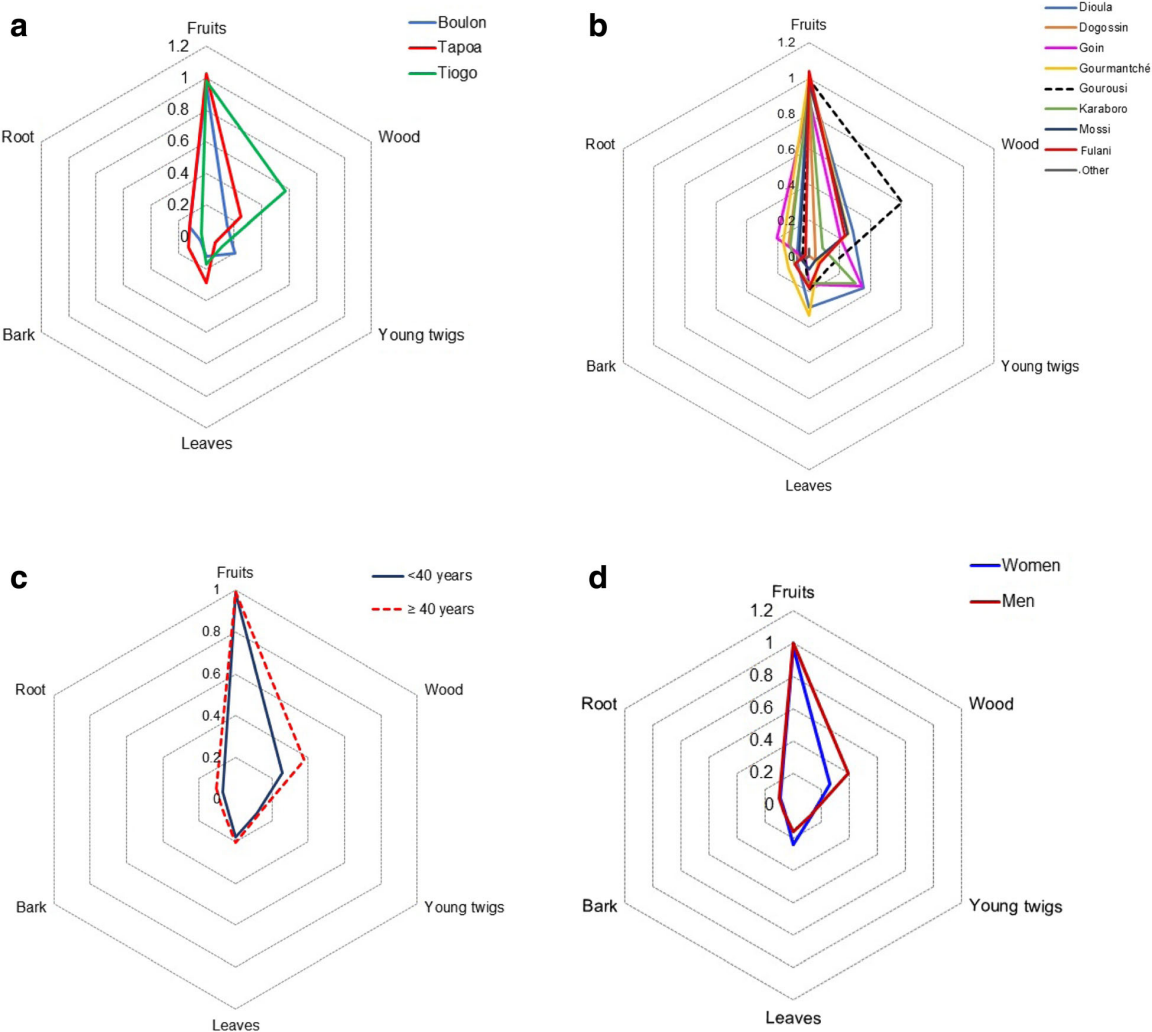
Radar chart showing the differences in the use of *Gardenia erubescens* plant parts between location (**a**), ethnic groups (**b**), generations (**c**), and gender (**d**)

Knowledge on the use of *G*. *erubescens* varied greatly across the plant parts (Fig. 5). Irrespective of the examined factors, fruit was the most used, followed by wood, leaves, and young twigs (Fig. 5a, b). The least used parts of the plant are the bark and roots. It was found that all ethnic groups use the fruit. Furthermore, the leaves were commonly used by Gourmantché, Dioula, Gourounsi, and Fulani while the wood was mainly used by Gourounsi (Fig. 5b). Aside from the use of its fruits, respondents below 40 years have low use value for the leaves, bark, and roots (Fig. 5c). Knowledge on fruit use was similar across the three study areas—Tapoa-Boopa, Boulon, and Tiogo (Fig. 5a). Regarding the wood of *G*. *erubescens*, respondents in Tiogo, belonging to the ethnic group of Gourounsi with an age above 40 years, reported more uses than others (Fig. 5). Regarding gender, men reported more uses based on their traditional knowledge when compared to women irrespective of the plant parts used with the exception of the leaves indicating a relatively higher UV for women (Fig. 5d).

### Cultural importance of *G. erubescens* by use categories

The cultural importance of *G*. *erubescens* varied significantly among ethnic groups and study sites (*p* < 0.05) in the following use categories: medicinal, construction, energy (use of wood as fuelwood), and cultural use (see Table 4). In addition, the cultural importance of other categories of use (food, fodder, and magic) was statistically similar between ethnic groups and sites (Table 4). Table 5 revealed that the importance of *G*. *erubescens* varied significantly among generation for the category for medicinal uses only while the importance of other categories of use was statistically similar between generations.

**Table 4:** Mean values of the cultural importance of *G*. *erubescens* use-category by site and ethnic groups

Use-category	Site			Ethnic group		
	df	*H*	*p* value	df	*H*	*p* value
Food	2	2.00	0.37 ^ns^	7	10.74	0.15 ^ns^
Medicine	2	15.38	< 0.001***	7	34.22	< 0.001***
Construction	2	8.49	0.014*	7	30.31	< 0.001***
Fodder	2	3.93	0.14 ^ns^	7	4.69	0.69 ^ns^
Energy	2	40.84	< 0.001***	7	50.04	< 0.001***
Cultural	2	32.31	< 0.001***	7	55.64	< 0.001***
Magic	2	5.90	0.052 ^ns^	7	5.21	0.63 ^ns^

*** = *p* < 0.0001; ** = *p* < 0.001; * = *p* < 0.01; ns = *p* > 0.05; *df* degree of freedom, *H* Kruskal-Wallis statistic, *p* value probability value computed

**Table 5:** Mean values (and standard error) of the cultural importance of different parts of *Gardenia erubescens* by generations

Generation	Food	Medicine	Construction	Fodder	Energy	Cultural	Magic
< 40 years	2.90 (0.54)	0.48 (0.88)	0.07 (0.35)	0.01 (0.07)	0.28 (0.61)	0.16 (0.45)	0.00 (0.00)
≥ 40 years	2.89 (0.54)	0.72 (0.98)	0.04 (0.27)	0.00 (0.00)	0.30 (0.57)	0.17 (0.41)	0.01 (0.09)
Mann-Whitney test	30135^ns^	26363**	30484^ns^	29956^ns^	28862^ns^	29142^ns^	29690^ns^

*G*. *erubescens* was more important (higher IP) in Tiogo (4.32 ± 0.39) than in Tapoa-Boopo (4.06 ± 0.40) and Boulon (3.75 ± 0.38); more important for Gourounsi (4.41 ± 0.39), Dioula (4.21 ± 0.42), Gourmantché (4.17 ± 0.41), Goin (4.11 ± 0.36), and Fulani (3.80 ± 0.41) than other ethnic groups; and slightly more important for older informants (4.14 ± 0.40) than younger informants (3.90 ± 0.38).

**Fig. 6:**
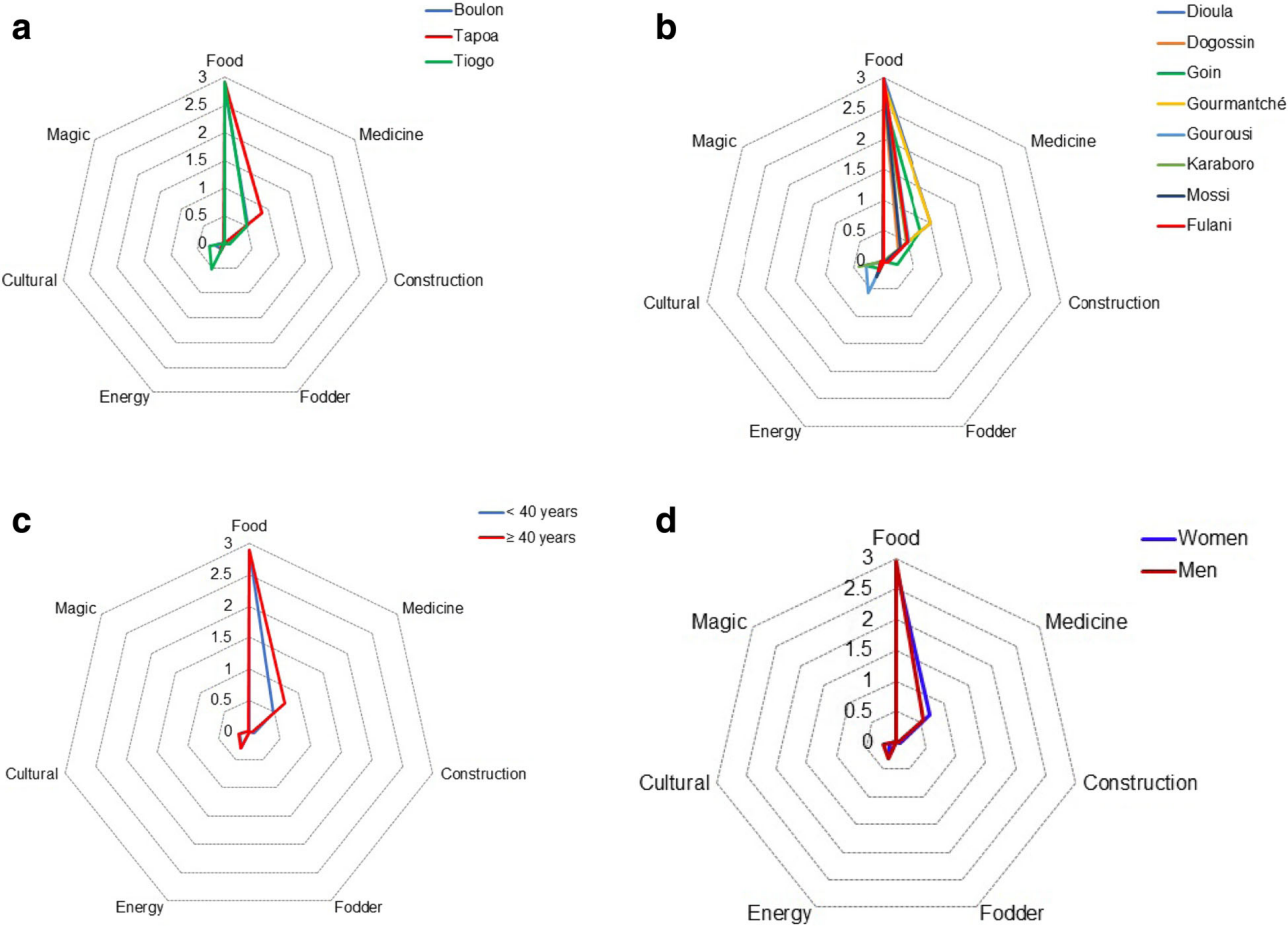
Radar chart showing the differences in the use of *Gardenia erubescens* plant parts across locations (**a**), ethnic groups (**b**), generations (**c**), and gender (**d**)

Irrespective of the predictors considered in this study (study site, ethnic group, and generation), food use value (2.89 ± 0.03) was the most important followed by medicine (0.63 ± 0.11), energy (0.29 ± 0.13), cultural (0.17 ± 0.07), construction (0.05 ± 0.02), magic (0.005 ± 0.006), and fodder (0.003 ± 0.00). With respect to the study sites, traditional knowledge revealed that food use values were more important in the three study sites (Fig. 6), while medicinal uses were important in Tapoa-Boopo, and energy (fuelwood) in Tiogo (Fig. 6a). Considering the ethnic groups, Gourmantché and Goin gave more importance to food use while the Gourounsi gave more importance to the energy use compared to other ethnic groups (Fig. 6b). In relation to generations based on their TK, food, medicinal, and cultural uses were more important for older informants than younger informants while the use of the species for construction was more important for younger informant than older informants (Fig. 6c, Table 5). With respect to gender, food, cultural, and magical uses were more important for men than women while energy and medicinal uses were more important for women than men (Fig. 6d).

## Discussion

The recorded total of seven use-categories as food, medicine, construction, fodder, energy, cultural, and for magical purposes is an indication that *G*. *erubescens* is an important plant species for nutrition and livelihood options across the three case study areas. Therefore, *G*. *erubescens* is among the plant species that have multiple uses similar to other species such as *Vitellaria paradoxa*, *Parkia biglobosa*, and *Adansonia digitata* that are commonly found in the parklands in Burkina Faso as indicated in a previous study [2]. Despite the importance and multipurpose nature of tree species in the Sahel and Burkina Faso in particular, traditional knowledge on their uses are not evenly distributed even among members in a given community. Therefore, this study was based on the calculation of important ethnobotanical indices (ethnobotanical use value and cultural importance index) in order to evaluate the level of knowledge of *G*. *erubescens*’ uses according to study sites, ethnic groups, generations, and gender. These indices have already been the subject of two previous studies conducted in the sub-region [32, 33]. It was found that traditional knowledge and cultural importance of *G*. *erubescens* varied significantly across category of use.

### TK on the use of *G*. *erubescens*

The overall knowledge of *G*. *erubescens* uses varied greatly across ethnic groups due to the cultural heritage, knowledge being transmitted from generation to generation within the same ethnic group. In this regard, *Gourounsi*, *Goin*, and *Gourmantchés*’*s* knowledge was relatively higher than that of other ethnic groups, confirming that cultural differences determine plant use habits—a view expressed by other studies in West Africa [34–36]. Furthermore, indigenous ethnic groups for example the Gourounsi had more knowledge on the use of the wood of *G*. *erubescens* compared to other ethnic groups. This is because this group has lived in these communities for centuries and closely interacting with their environment, which has contributed to a gradual accumulation of knowledge over time. Thus, ethnicity constitute an important factor on the uses and knowledge of plants between communities and our findings support the general trend that ethnicity influences traditional knowledge on plant use [2, 33, 34, 37]. Ethnicity according to Etongo et al. [2] goes beyond belonging to a cultural group or way of life, and includes cultural beliefs, taboos, rituals, and ideology of social groups. Therefore, groups that interact with the environment daily or more frequently such as cattle headers (*Mossi*) in search of fodder or traditional healers (*Gourmantché*) in search of herbs have relatively more knowledge on the use of different plant parts of *G*. *erubescens*.

Additionally, this study showed that the elderly generation had more local knowledge on the use of *G*. *erubescens* than the younger generation. This could be explained, on the one hand, by the fact that older people have more uses than the younger ones and secondly by the fact that it is the result of the transmission of knowledge across generations [9]. According to Geng et al. [38], the transmission of knowledge from the older to the younger generation is nowadays confronted with a gradual disappearance of these knowledge systems partly due to migration from rural to urban areas. Several other studies including those of Lougbégnon et al. [39] and Salako et al. [15] have shown that individual characteristics such as age, for example, can influence knowledge of resources and their use within a community. The difference observed in medicinal use of the plant can be explained by the fact that such uses are often specific and are mostly reserved for the elderly. Besides, the collection of medicinal plants is particularly accompanied by certain religious, ethnic, empirical, and magico-religious rules [40]. Therefore, elderly have more knowledge in this category of use than young people given that these restrictions that are culturally motivated are applicable only to the latter group. However, traditional knowledge on plant uses does not accumulate by default and even those in the older age category (> 40 years), needed to have interacted with the natural environment in their respective communities—a pre-condition for them to gain more knowledge than the than younger informants. These results are similar to those of Goudégnon et al. [9] who reported significant difference between generations and ethnic groups on *Lannea microcarpa* Engl. & K. Krause in Benin.

The most common medicinal uses are the treatment of gastric diseases and syphilis, the treatment of sexual asthenia and female infertility. The variation in food use could be explained by the fact that the local populations of the three study sites do not use the plant in the same way. For instance, according to the Non-Timber Forest Products Promotion Agency (NFPA), in some areas of Burkina Faso, including the province of Nayala, the fruit would be prepared in paste form such as *Tô* (local name of maize or millet paste) or in association with couscous (a North African dish of steamed semolina usually served with meat or vegetables). In other localities, the fruits are eaten raw or cooked [8].

The knowledge on food uses was similar among the study sites, ethnic groups, generation, and gender. Our finding can be justified by the fact that food uses are common among the socio-demographic attributes as reported by Goudégnon et al. [9], suggesting a consensus for *G*. *erubescens* food uses.

The results also showed that fruits were the most used plant part for food by the local populations. Several studies have reported the use of fruits in the food and medicinal uses in West Africa [3, 13, 18]. In addition to its fruits, leaves, roots, and young twigs were also used for medicinal purposes which give a considerable value to the species since most of these plant parts are used in several areas. This represents an asset to valorize the plant and its parts for income, food, and nutrition. The fact that fruits was the most important plant part is an evidence that *G*. *erubescens* is an indigenous food tree species.

### Cultural importance of *G*. *erubescens* by category of use

A study by Etongo et al. [2] stressed that understanding the cultural importance of plant resource is crucial for an informed management. The current study revealed differences in form of uses across the study sites, ethnic groups, and generation. Food and medicine were the most culturally important uses of *G*. *erubescens* in Burkina Faso. These use-categories are also the most important for *Bombax costatum* [41], *Lannea microcarpa* [9], and *Borassus aethiopum* [15]. Food and medicinal uses are basic needs for local population living in Boulon, Tapoa-Boopo, and Tiogo. These two use-categories are invaluable for local livelihoods especially with the relatively greater reliance of the rural communities on herbal/traditional medicine partly due to poverty and also due to the lack of health facilities that are of adequate standards to provide medical services to the rural population. The fact that food and medicinal uses were more important for women compared to men is an indication that women are responsible for kitchen and most often engaged in the marketing of food and medicinal products in the markets. Men on the other hand are often responsible for constructions, cultural, and magic care of the household members.

## Conclusion

The present study evaluated indigenous knowledge of the importance and use of *G*. *erubescens* in the villages bordering Boulon, Tapoa-Boopo, and Tiogo forests in Burkina Faso. The species is known by different local names depending on the ethnic groups present. The most used part of the species is the fruit, followed by the bark, wood, young twigs, roots, and leaves. *G*. *erubescens* is a multi-purpose species similar to most indigenous fruit tree species in West Africa and the Sahel in particular. Its ability to provide food and medicinal values makes it an important fruit tree species that deserves more attention in terms of exploitation and sustainable management than it is currently given especially as the species is considered threatened based on National Biodiversity Assessment. The interest in its fruits as a source of food supply by local population is an asset for the sustainable management and conservation of the species especially in this era of the Sustainable Development Goals that considers health and nutrition, food security, and environmental protection among its priority areas of intervention. In order to avoid the overutilization of the fruits of *G*. *erubescens*, it would be good to train women for good practices of fruits collection. Moreover, it will be important to put in place a national and special research and development program under the joint umbrella of the ministries of agriculture and scientific research sponsored by the government involving all the possible actors including researchers, developers, and producers. Effective propagation methods of the species can help for sustainable conservation and its domestication.

## Appendix

**Table 6:** Specific uses of *G*. *erubescens* in Burkina Faso

Plant parts	Use category	Processing method	Addition of ingredients	Form of use	Purpose of use	FL (%)
Fruits	Medicine	Roasted and crushed	Porridge, Shea butter	Drink the liquid, massage	Small buttons on the chest	8.33
		Boil in water	Spice	Drink the liquid, purge	Navel pain	8.33
		Boil in water	Butter of cow’s milk	Massage	Sprain	8.33
		Roasted and crushed	Shea butter	Application on the wound	Buttons all over the body	8.33
	Food	Remove the flesh	–	Drink the liquid	Human nutrition	93.35
		Boil in water the fruits	–	Drink the liquid	Human nutrition	8.2
		Cook in paste	Couscous	Drink the liquid	Human nutrition	30.07
Leaves	Medicine	Boil in water	–	Take a bath	sore nerve	8.33
		Boil in water	–	Drink the liquid, take a bath and purge	Premature infant	16.67
		Boil in water	Sugar	Drink the liquid	Lack of appetite	16.67
		Boil in water	–	Drink the liquid	Diabetes	16.67
		Boil in water	–	Drink the liquid	Dysentery	8.33
		Boil in water	–	Drink the liquid, purge	Navel pain for adults	16.67
		Boil in water	–	Drink the liquid, take a bath and purge	Fragile child	8.33
		Boil in water	Shea butter	Drink the liquid, take a bath, massage	Navel pain for children	8.33
		Boil in water	–	Drink the liquid, take a bath and purge	Headache	8.33
	Fodder	Eat	–	Drink the liquid	Animal nutrition	0.39
Wood	Magic	Lay the branch or the wood at the entrance of his house	–	–	Expel someone from a village	38.48
	Energy	Collect dry wood	–	Fire wood	Cooking	22.07
	Construction	Collect branches	–	–	Fence of animal pens	2.74
Young twigs	Medicine	Boil in water	–	Take a bath	Delay of the first steps in children	83.33
	Magic	Attach to the door of the house	–	–	Protection against geniuses (spiritual)	6.83
		Lay behind his vehicle	–	–	Protection against accidents	3.9
		Lay the branch or the wood in the farm or on wood	–	–	Protection of farms and bundles of wood against thieves	32.42
		Lay the branch or the wood at the entrance of his house	–	–	Expel someone from a village	35.17
	Cultural	–	Clay	–	Construction of wood fireplace for cooking	4.1
		Attach two twigs in the shape of a cross	African fan palm leaf basket	–	Indicate the death of a young lady	5.66
Bark	Medicine	Boil in water	–	Drink the liquid	Lack of appetite in children	8.33
		Crush dry bark	–	Drink the liquid	Stomach aches	8.33
		Boil in water	–	Drink the liquid, purge	Fragile child	8.33
	Magic	Crush dry bark	–	–	Protection against geniuses, helps kill big game in the bush	31.64
Roots	Medicine	Roasted and crushed	Cow milk	Rub the body	Sore nerve	25
		Boil in water	Butter of cow’s milk	Take a bath, massage and purge	Delay of the first steps in children	83.33
		Boil in water	–	Drink the liquid, purge	Ulcer	8.33
		Boil in water	–	Drink the liquid, take a bath	Premature infant	25
		Boil in water	Shea butter	Drink the liquid, take a bath and massage	Sore nerve	25
		Boil in water	Shea butter	Drink the liquid, take a bath and massage	Navel pain for children	25
		Boil in water	–	Drink the liquid, take a bath	Headache	8.33
		Roasted and crushed	Butter of cow’s milk	Massage	Back pain	8.33
		Roasted and crushed	Butter of cow’s milk	Massage	Muscle aches	8.33
Seeds	Medicine	Boil in water or Roasted and crushed	Butter of cow’s milk	Massage	Muscle aches	16.67
		Boil in water	–	Drink the liquid, purge	Navel pain for adults	8.33
		Roasted and crushed	Butter of cow’s milk	Massage	Back pain	8.33
Leaves + roots + fruit	Medicine	Boil in water	–	Drink the liquid, take a bath	Premature infant	8.33
		Boil in water	–	Drink the liquid, take a bath, purge	Navel pain	8.33
